# Small-angle X-ray scattering method to characterize molecular interactions: Proof of concept

**DOI:** 10.1038/srep12085

**Published:** 2015-07-10

**Authors:** Nicholas Allec, Mina Choi, Nikhil Yesupriya, Brian Szychowski, Michael R. White, Maricel G. Kann, Elsa D. Garcin, Marie-Christine Daniel, Aldo Badano

**Affiliations:** 1 Division of Imaging, Diagnostics, and Software Reliability, Office of Science and Engineering Laboratories, Center for Devices and Radiological Health, U.S. Food and Drug Administration, Silver Spring, Maryland, USA; 2 Fischell Department of Bioengineering, University of Maryland, College Park, Maryland, USA; 3 Department of Chemistry and Biochemistry, University of Maryland, Baltimore County, Maryland, USA; 4 Department of Biological Sciences, University of Maryland, Baltimore County, Maryland, USA

## Abstract

Characterizing biomolecular interactions is crucial to the understanding of biological processes. Existing characterization methods have low spatial resolution, poor specificity, and some lack the capability for deep tissue imaging. We describe a novel technique that relies on small-angle X-ray scattering signatures from high-contrast molecular probes that correlate with the presence of biomolecular interactions. We describe a proof-of-concept study that uses a model system consisting of mixtures of monomer solutions of gold nanoparticles (GNPs) as the non-interacting species and solutions of GNP dimers linked with an organic molecule (dimethyl suberimidate) as the interacting species. We report estimates of the interaction fraction obtained with the proposed small-angle X-ray scattering characterization method exhibiting strong correlation with the known relative concentration of interacting and non-interacting species.

The ability to quantitatively characterize interactions between biomolecules including proteins, nucleic acids, polysaccharides, lipids, hormones, and vitamins is essential for understanding the molecular basis of biological processes, for improving the design of high-specificity pharmacological approaches, for the immediate assessment of response to therapy, and for the identification of relevant disease mutations^1^. Protein-protein interactions (PPIs) in particular are fundamental in biological processes from the formation of cellular structures and molecular machinery, to signal transduction and membrane diffusion. A better understanding of PPIs would allow for more accurate predictions of protein cellular function including those involved in diseases and therapeutical approaches. The method presented here improves on existing techniques by addressing several key challenges for the accurate identification and quantification of biomolecular interactions.

Existing methods for detecting PPIs include the yeast-two-hybrid (Y2H) method^2^, fluorescence resonance energy transfer (FRET)^3^, bioluminescence resonance energy transfer (BRET)^4^, and positron-emission tomography (PET)^5^. The Y2H system is one of the most widely used methods for detecting PPIs. Of the 6,000 PPIs detected in humans in 2005, about half of the data came from the Y2H method^2^. This *in vitro* technique, however, has a notoriously high possibility for false positives attributed in part to localization of the complex in a compartment different from the protein’s natural cellular environment and proteins that overcome nutritional selection in yeast. In addition, any protein interaction proven to work in yeast may not accurately reflect an interaction taking place in a foreign protein’s native cellular environment in other organisms^6^. This is not the case for FRET and BRET. These two methods have been shown to work *in vivo* and like Y2H, are used widely. As optical approaches, however, signal attenuation by tissue limits the techniques to superficial lesions and small animals where they have exhibited low sensitivity^4^. Characterization of PPIs using PET does not suffer from such limitation^5^. This technique can localize PPIs *in vivo* deep in the body, although only a single example of its implementation has been reported^7^. A disadvantage of PET is the use of a radioactive tracer, with short half-life, thus limiting imaging time. Aside from experimental PPI detection and imaging, available methodologies for mapping protein interaction networks based on computational methods supported by experimental observations have had limited extent and low specificity. In humans, these networks are especially limited with only 10% of predicted interactions corroborated in the literature^8^.

We propose and investigate the feasibility of using small-angle X-ray scattering (SAXS) for characterizing biomolecular interactions. SAXS is a high resolution characterization technique able to resolve features in the range between 1 and 100 nm. An important benefit of using this technique is that biological specimens can potentially be studied in their natural environment. Typical uses for SAXS are in the determination of shape, size, distributions, and locations of various nanostructures. Tagging biomolecules with high contrast materials (such as gold nanoparticles) led to the formation of useful molecular rulers^9^,^10^,^11^. The use of heavy atom labels to determine characteristic distances in particles was previously described by Feigin and Svergun^12^. SAXS signatures have also been used as an indication of interparticle distance for GNPs assembled in an ordered fashion^13^,^14^ as well as for tumor imaging^15^ and tissue characterization/differentiation^16^,^17^,^18^,^19^. Although this technique has been instrumental in the characterization of biomolecules, remaining challenges include low contrast and high background noise^20^.

The proposed methodology requires highly scattering molecular probes that selectively bind with high affinity to targeted biomolecules involved in putative interactions. When the targeted biomolecules come in close proximity (e.g., 1–100 nm), the probes, which are of sufficiently small size to prevent perturbing the system and avoid steric effects, provide a characteristic scattering signature that depends on intermolecular distance and is greater in intensity than that of the targeted molecules. The probes, therefore, provide SAXS signatures that are indicative of the interaction between two targeted biomolecules within the specimen (Fig. 1a,b). We define the interaction fraction, 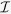, as the ratio of the concentration of biomolecular interactions to the sum of the concentration of interacting and non-interacting labelled biomolecules in the examined volume. The estimate of 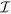 will be affected by several environmental factors including conditions that prevent the labelled species from physically reaching each other. For binary detection tasks (interacting/non-interacting), a threshold interaction fraction can be implemented. The described method differs from techniques that only detect interacting populations (such as PET) in that information regarding both interacting and non-interacting species are obtained. The probes are designed to elastically scatter X rays at small angles above the background signal from the biomolecules to which they are bound. For detecting the interaction of two or more biomolecules, several different probes can be used. For example, the probes could vary in size (e.g., 5 and 10 nm), shape (e.g., spherical and rod-shaped), and/or base material (e.g., gold and silver). The proposed method has the potential to provide increased spatial resolution and higher specificity while allowing for deep tissue imaging, complementing other methods under development for biomolecular interaction detection including FRET, BRET, and PET, potentially overcoming some of the existing challenges.

We describe a proof-of-concept demonstration of the technique using gold nanoparticles (GNPs) as molecular probes. In SAXS, the scattered X-ray intensity is measured as a function of the scattering vector, *q*, which is related to the scattering angle, 2*θ*, by *q* = 4*π*sin(*θ*)/*λ*, where *λ* is the incident radiation wavelength. The coherent particle scattering intensity, *I*(*q*), within the *q* range of 0–3 nm^−1^ (where the angular dependence of the atomic form factor can be neglected)^21^ is proportional^22^ to *NV*^2^(Δ*ρ*)^2^*F*(*q*), where *N* is the number of particles, *V* is the particle volume, *F*(*q*) is the particle form factor, and Δ*ρ* is the difference in electron density of the particle and background (*ρ* ∝ Z*C* assuming uniform electron density, where Z is atomic number, and *C* is atomic density). GNPs are thus highly suitable as molecular probes due to the high Z of gold (79) compared to lower Z atoms typically found in biomolecules and the high atomic density of GNPs (5.9 × 10^22^ atoms/cm^3^)^23^, with an electron density one to two orders of magnitude higher than that of proteins. The suitability of GNPs as molecular probes for our method is further enhanced by their inertness, tunable size, and availability of surface modification techniques with a variety of ligands^24^,^25^,^26^,^27^,^28^,^29^.

As part of the proof-of-concept model system, we use dimerized GNPs to represent the interacting species. The preparation and study of GNP dimers in general remains a challenge that only few groups have been tackling. Alivisatos^30^ and Mirkin^31^ have been the pioneers in this work, using DNA to link nanoparticles, while Novak and Feldheim^32^ have assembled GNPs into dimers using molecular links. Hofmann *et al.*^33^ used a modified solid phase approach to obtain dimers. In general, the yield of dimerization remains low, the characterization techniques available have some limitations, due in part to the large flexibility of the dimers formed. Thus, at this point we have opted for an improved dimerization method that relies on (*i*) using a very stable nanoparticle coating (thiolated PEG) with a thickness corresponding to half of the desired interparticle distance, (*ii*) functionalizing only about 10% of the nanoparticle surface coating termini, and (*iii*) using a very short crosslinker (dimethyl suberimidate, DMS) for homo-dimerization. The thick PEG coating allows for both water solubility and decreased flexibility of the dimer. The relatively large interparticle distance created this way mimics well the possible distance range expected during PPI detection. To ensure stability of the nanoparticles during intracellular detection of PPIs, thioctic acid-derived ligands (divalent thiols) for their coating can be used^33^,^34^.

## Methods

**Figure 1:**
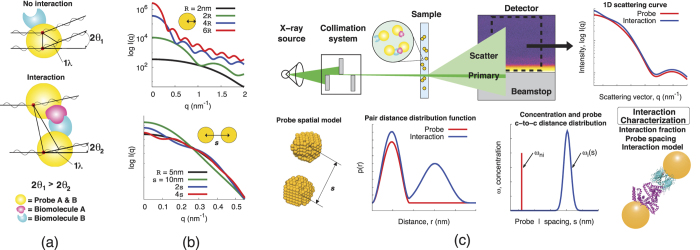
The SAXS patterns of suspected interacting biomolecules are analyzed to characterize the interaction fraction. (**a**) Interacting and non-interacting biomolecules tagged with targeted probes provide different coherent scattering signals (where *λ* is the wavelength of incident radiation). (**b**) Theoretical scattering curves of (top) monomer spheres^45^ with radius *R* and (bottom) dimers^44^ with center-to-center spacing, *s*, with a scaled monomer curve for comparison, showing characteristic features when probes are in close proximity. (**c**) Measurement setup illustrating a line collimated (Kratky-style) SAXS system as used in this study. The pair distance distribution function, concentration *s* distribution, and spatial model of the probes are determined from data analysis (theoretical results shown here). The interaction fraction, 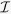, the probe spacing distribution, and the spatial interaction model are extracted from the data to characterize the interaction.

We summarize the method in Fig. 1c. Targeted probes are attached to biomolecules of interest. The SAXS profile, *I*(*q*), of a solution containing biomolecules that can interact is measured using laboratory or accelerator instrumentation. Neglecting the effect of scatter from biomolecules, the scattering intensity is the sum of the contributions from probes bound to non-interacting (ni) and interacting (i) biomolecules as follows,
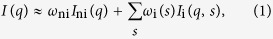
where *ω* is the concentration of populations, which encompasses non-interacting particles and interacting particles with a distribution of probe center-to-center spacings, *s*. The scatter pattern is analyzed to obtain the pair distance distribution function, *p*(*r*), which represents the electron-density-weighted distribution of distances, *r*, between pairs of points in the particle, and the concentration distribution function, *ω*. Determining *ω*_i_(*s*) provides additional spatial information on the interaction. However, *ω*_i_(*s*) carries additional complexity in cases where more than two probes are involved in the interaction of interest (e.g., if one or more of the biomolecules has multiple active binding sites) where the distribution peaks become less distinct and the analysis becomes less definitive. It is helpful, though not necessary, to have *a priori* information of the putative interacting system tested including biomolecule size and number of binding sites to help guide data analysis. The derived quantities include the probe spacing distribution, a spatial model of the interaction, and the interaction fraction:
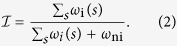
The probe spatial model (e.g., bead model^35^) can be complemented by biomolecular models^36^ for additional insight.

To demonstrate the proof-of-concept, a model system was developed based on GNPs (10–17 nm in diameter) coated with 10% of HSPEGNH_2_ and dimerized using DMS in water, representing a single binding site. The model system was tested under different volumetric ratios of monomers and dimers. In addition to measurements of the model system in a water background, a cell lysate background was used to emulate the type of scattering conditions that would be encountered in a cellular environment. First, monomers were mixed directly with lysate and characterized using SAXS. Then, due to competition of the lysate’s reducing agent with thiolated PEG used to link the dimers, monomers and dimers were characterized using a separate capillary for lysate (acting as background).

### Sample preparation

**Figure 2:**
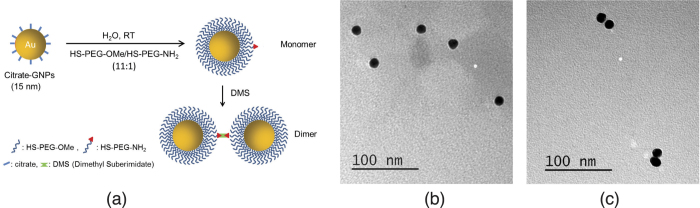
Interaction model system. (**a**) Schematic representation of the synthesis of the model system. TEM image of (**b**) monomers and (**c**) dimers, representing non-interacting and interacting species, respectively.

Approximately 15 nm diameter (estimated from Transmission Electron Microscope, TEM, images) GNPs were synthesized following the Frens-Turkevich method^37^,^38^. A 100 ml solution of 0.6 mM HAuCl_4_ was heated to reflux. Then 2.5 ml of 5% sodium citrate was added and the mixture was stirred for 20 min. GNP probe sizes were measured using Dynamic Light Scattering (DLS). GNP dimers were prepared by functonalizing probes with HS-PEG-NH_2_ and then linking them via 1.1 nm long DMS (Pierce Biotechnology Inc., Rockford, IL, USA) (Fig. 2). Solutions of HS-PEG-NH_2_ and HS-PEG-OMe were prepared by dissolving 0.008 g and 0.050 g, respectively, in 10 ml of water. 2.34 ml of the HS-PEG-NH_2_ solution and 4.68 ml of the HS-PEG-OMe solution were premixed, then added to 80 ml of GNP probe solutions. The mixture was stirred for approximately 4 hours. Ligand exchange was confirmed using DLS to observe a change in size and zeta-potential. A total of 0.0021 g DMS was dissolved in 1 ml of triethanolamine buffer solution (pH 8) and then 0.156 ml of this solution was added to a 5 ml solution of the functionalized GNPs. The mixture was stirred overnight. Formation of dimers was observed by DLS. Five samples were prepared: a probe solution, a dimer solution, and three mixtures of the probe and dimer solutions with differing volume fractions of dimer solution (0.33, 0.50, and 0.66). The probe solution was diluted until the scattering intensities observed in SAXS matched the scattering intensities measured from the dimer solution in scattering angles indicative of a spherical particle (approximately 0.2 to 0.6 nm^−1^). Once the appropriate dilutions were achieved, the dimer and probe solutions were mixed.

The lysate consisted of E. coli BL21(DE3)pRILP cells from a cell pellet of 16.61 g suspended in 50 ml lysis buffer. The lysis buffer consisted of 20 mM Tris pH 8.0, 30 mM NaCl, 1 mM DTT, 0.1% Tween 20, 25 mg lysozyme (MP Biomedicals, 4 *μ*l), benzonase (Sigma-Aldrich, 250 U/*μ*l), EDTS-free cOmplete protease inhibitor cocktail tablet (Roche). The cells were first suspended in the lysis buffer by repeated pipetting. The solution was then sonicated using a Q Sonica Q700 sonicator. This cell lysate was then diluted 1:2 (lysate to water) for scattering experiments. For the measurements with monomers mixed directly with lysate, three solutions were prepared: undiluted monomers in water, a 1:1 mixture of monomers and lysate, and a 1:1 mixture of diluted monomers (in water) and lysate which led to a final monomer concentration of 1:19. For the measurements with monomers/dimers in a separate capillary, the monomers and dimers were kept in a water solution (similar to the water background experiments) in a capillary separate from the lysate.

### SAXS measurements

A SAXSpace system (Anton Paar, Ashland, VA, USA) was used for all SAXS measurements. The instrument, which uses Cu K_*α*_ radiation (*λ* = 0.154 nm), was configured in Kratky block line collimation mode with an accessible *q* range of 0.0732–1.66 nm. The system is equipped with a CCD camera with a pixel pitch of 24 *μ*m in an array of 2084 × 2084 pixels. The camera uses a Gd_2_O_2_S:Tb phosphor screen matched to 8-keV X rays. Samples are loaded into the system via a 1-mm diameter quartz capillary positioned at a distance of 305.3 mm from the CCD and temperature-controlled at 18 °C. The collimation system, sample chamber, and beam path were enclosed in vacuumed space with a pressure below 3 mbar. The CCD pixels were binned along the length of the beam (2 cm). For each measurement, 2400 frames were obtained at 1 s exposures and averaged. A beam profile, dark, buffer, probe, dimer, and three mixtures of probe and dimer (0.33, 0.50, and 0.66 volume fraction of dimer solution) measurements were acquired on the same day. The buffer (deionized water) was measured under the same conditions as the sample.

For measurements of the monomers mixed directly with lysate, the SAXS settings were adjusted to an exposure time of 2.75 s and minimum of 200 frames. The buffer consisted of lysate and deionized water (replacing the monomer solution) and was measured under the same conditions as the sample. In order to confirm that the dimers could be observed in a lysate background, modifications were made to the scattering measurement since the reducing agent in the buffer used to lyse the cells (DTT) competes with the thiolated PEG used to link the dimers. An implementation was constructed that did not require direct mixing of the dimers with lysate. The cell lysate was sealed with wax in a 1 mm outer diameter, 0.01 mm wall thickness quartz capillary (from Hampton Research, Aliso Viejo, CA, USA) and was positioned in the X-ray beam path, between the original capillary cell holder and the CCD camera. The lysate capillary was placed approximately 6 mm from the monomer/dimer solution. The buffer consisted of the same setup, but the monomer/dimer solution was replaced by deionized water. For lysate measurements, the SAXS settings were adjusted to an exposure time of 6 s and minimum of 150 frames. The exposure time was increased due to the increased scattering intensity of the lysate background. The capillary temperature was reduced to 14 °C.

### Alternative characterization methods

Aside from SAXS, DLS and TEM were also used for sample characterization. A Malvern Zetasizer Nano ZS (Worcestershire, UK) was used for DLS. Three measurements were acquired for 1 ml of each sample and averaged. The size estimates were obtained by number %. A JEOL JEM-1400 (Peabody, MA, USA) was used for TEM at a voltage of 80 kV. A 10-*μ*l droplet of sample solution was placed on an Electron Microscopy Sciences (Hatfield, PA, USA) 200-mesh copper grid with carbon film.

### Data analysis

Scatter patterns were acquired using the SAXSdrive software (Anton Paar, Ashland, VA, USA) and data analysis was performed using custom Matlab and C++ codes. The sample and buffer solution scatter curves were subtracted by dark measurements, followed by scaling (if necessary) and subtraction of the buffer from the sample scatter. The indirect Fourier transform (IFT) method^39^ was implemented with 20 splines between 0 and *D*_max_, which is defined as the *a priori* estimate of the longest pair distance in the particle. *D*_max_ was initially estimated and then adjusted until the *p*(*r*) shown near *D*_max_ did not descend sharply, go negative, or oscillate^40^. We used 29 different stabilization values, *α*, of 10^*n*^ for *n* = −4, −3.5, −3, …, 10 which we found to be a sufficient range in finding an appropriate *α* and set of weights. The appropriate *α* chosen was determined by following procedures outlined by Glatter *et al.*^39^ The *α* for the probe solution was 5, and ranged between 2.5 and 4.5 for the dimer solution and mixes. Bead models of selected scattering curves were obtained using the ATSAS software package^35^,^41^. Each model was generated from 20 runs of DAMMIF^42^, which were averaged using DAMAVER^43^.

**Figure 3:**
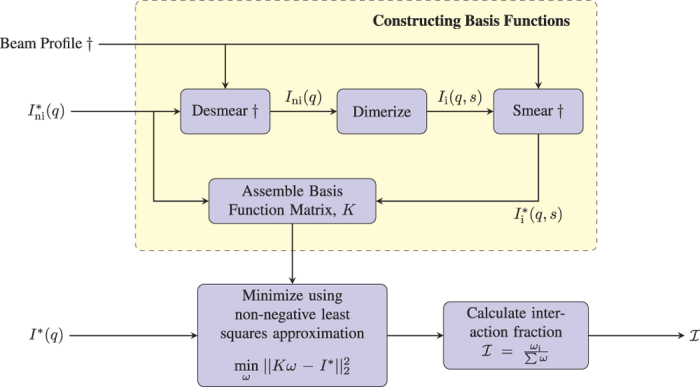
Flow diagram of the data analysis procedures. The superscript * is used to indicate smeared data. Steps that include a † are only necessary for a line-collimated SAXS system and can be ignored when using point-collimation.

For our model system, the interaction fraction (Eq. (2)) is equivalent to the concentration of dimers over the sum of dimers and monomer probes. The concentrations are estimated by fitting the measured scatter profile of the system of interest to a series of basis scatter functions, as will be described below. A general overview of the steps involved are shown in Fig. 3. First, the measured scatter from probes was fitted and desmeared using the IFT method to determine *I*_ni_. A set of basis scatter functions (for different *s*) were then calculated using the following equation^44^,
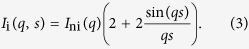


The range of *s* was chosen to be between 17 nm (*s*_min_ = 2 × *D*_max_ of the probe, which is the minimum spacing achievable assuming the probes cannot overlap in space) and 50 nm (*s*_max_) in steps of 1 nm.

Smearing was applied to the basis functions to be compatible with the measured scatter from the sample of interest, which was also smeared. The smearing was applied as follows^45^,
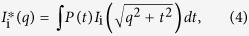
where the superscript * is used to denote smeared functions. *P*(*t*) is the trapezoidal fit of the beam length profile and *t* is in terms of nm^−1^. The basis functions were then assembled into a matrix,
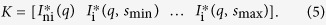


The weights for probe, dimer, and mixed sample measurements were solved using a non-negative least squares approximation^46^ based on Eq. (1),
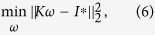
where I^*^ is the measured scatter intensity, all weights are greater than or equal to 0, and
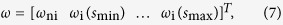
where superscript *T* indicates the transpose. Equation (6) was fit over a *q*-region from 0.0732–0.39 nm^−1^ with a Δ*q* of 0.0032. This range of *q* was selected because it contained the differentiating feature in the scatter profile indicative of dimers. Alternatively to the method discussed herein, existing methods^47^ for analyzing mixtures of scattering intensities could be leveraged to determine *ω*.

After determining the weights, the interaction fraction for the sample of interest was calculated from Eq. (2). To determine the standard deviation of interaction fraction estimates, scatter profiles were measured five times for three solutions: a probe solution, a 0.5 dimer volume fraction mixed solution, and a dimer solution. The standard deviation (±1) was calculated from the resulting interaction fractions of the five measurements.

A fit of the interaction fraction as a function of dimer solution volume fraction (*x*) was determined as follows:
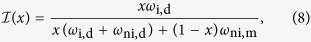
where it was assumed the monomer solution (subscript ‘m’) contained only monomers and the dimer solution (subscript ‘d’) contained a mix of monomers and dimers. This fit accounts for possible differences in concentrations of the monomer and dimer solutions. The fit and coefficient of determination, *R*^2^, were determined using the curve fitting toolbox in Matlab.

## Results

**Figure 4:**
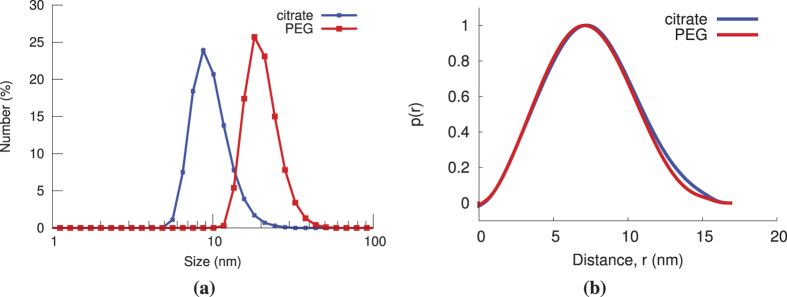
(**a**) DLS and (**b**) SAXS results for a batch of citrate and PEG coated GNPs. The average particle size, polydispersity index (PDI), and Z-avg from DLS were 10 ± 3 nm (21 ± 5 nm), 0.281 ± 0.021 (0.127 ± 0.017), and 20.78 ± 1.61 (29.45 ± 0.60 nm), respectively for the citrate- (PEG-) coated GNPs. The Z-avg value is influenced by larger aggregates in the sample and yields a higher average than that obtained by number %. The difference in size between the two particles is 11 nm. From SAXS, the diamater was determined to be 17 nm (from *D*_max_) for both sets of GNPs.

**Figure 5:**
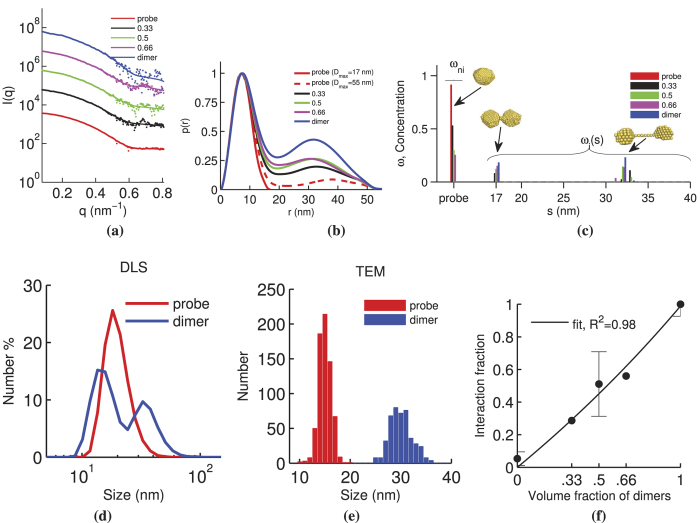
Proof-of-concept experimental results for the interaction model system corresponding to five samples with different volume fractions of dimer solution (0.00, 0.33, 0.50, 0.66, and 1.00). (**a**) 1D SAXS scatter plot of data (points) with approximated fits (solid lines). Each curve is offset by a factor of 10 for clarity. (**b**) *p*(*r*) derived from (a) computed with an estimated maximum distance parameter (*D*_*max*_) of 17 and 55 nm, the latter for comparison with mixtures and dimer *p*(*r*). (**c**) Concentration of non-interacting (*ω*_ni_) and interacting model species (*ω*_i_) derived from (a). Bead models of three selected basis functions (*I*_ni_(*q*), *I*_i_(*q*,17), and *I*_i_(*q*,32)) are shown at their respective spacing locations on the *ω* distribution. The range of *s* used for basis functions was from 17 to 50 nm. (**d**) DLS results from probe and dimer solution. **(e)** TEM histogram for probe diameters and maximum dimer dimensions. (**f**) Interaction fraction, 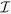, for five samples with differing volume fractions of dimer solution and the fit from Eq. (8).

The distance between GNPs in the dimer was predicted to be approximately 12 nm, which is within the size range of relevant biomolecular interactions^48^. The estimate of distance between GNPs is based on DLS data from the monomer that indicate a PEG thickness of approximately 5.5 nm around the gold core (Fig. 4), and the DMS linker which is around 1.1 nm in length. Mixtures with differing volume fractions of dimer solution were measured and analyzed. Although samples for typical SAXS experiments are monodisperse, mixtures are used herein to mimic the copresence of interacting and non-interacting biomolecules. Figure 5 shows the analysis chain and resulting interaction fraction for 5 samples prepared with different volume fractions of dimer solution: 0.00 (probe), 0.33, 0.50, 0.66, and 1.00 (dimer). It should be noted that the dimer solution was not assumed to contain dimers exclusively.

TEM and DLS were used as reference methods to provide comparison for results obtained with the proposed method. Although these methods provide complementary information, they are not suited for detecting *in vivo* biomolecular interactions. TEM cannot be used for *in vivo* applications or dynamic imaging in real time since it requires the sample to be either frozen (cryo-TEM) or dried. It is also not suitable for determining quantitative estimates of the population of interacting and non-interacting species. DLS assumes spherical particles and typically overestimates feature sizes since it measures the hydrodynamic radius^49^. This can be seen in Fig. 5 where the particle size determined from DLS was 33% larger than estimates from SAXS (DLS: 20 ± 5 nm, SAXS: 15 nm from Guinier plot analysis using spherical model). The TEM results showed an average particle size of 15 ± 1 nm (from 687 particles). It should be noted that the DLS data includes the contribution of the PEG shell to the GNP size estimate, whereas the SAXS and TEM data are heavily weighted by the gold core. The estimate of the maximum characteristic dimension of the dimer by DLS, SAXS, and TEM was 36 ± 12 nm, 47 nm, and 30 ± 2 nm (from 111 dimers), respectively. The SAXS estimate, 47 nm (= 32 + 15 nm), was determined from the most frequent solution of *ω* basis function fits (Fig. 5c) using a *D*_max_ of 55 nm from *p*(*r*) analysis (Fig. 5b).

The DLS estimate has limited reliability due to inaccuracies when measuring samples containing particles of different sizes^50^. The estimate from TEM is significantly shorter due to dehydration of the linker during sample preparation. Although the maximum dimension estimate from SAXS was 55 nm, a breakdown of the constituents of the dimer sample, as shown in Fig. 5c, shows a prominent population of dimers with a center-to-center spacing of 32 nm. Since the spacing of 32 nm includes two radii (i.e., 2 × 7.5 = 15 nm) of the two GNPs, it means that the distance between the surface of the two GNPs is about 17 nm. This is consistent with the 12 nm spacing estimated from the DLS data of the monomer, considering that the crosslinking of the two PEG ligands on each GNP most likely have induced them to stretch. There is little variation in *s* due to the limited flexibility in the small linker attached to the densely packed PEG coating.

The interaction fraction 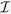, derived from *ω* in Fig. 5c, is shown in Fig. 5d where significant correlation between 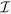 and volume fraction of dimers can be seen. Using the fit of Eq. (8), the values of *ω*_i,d_, *ω*_ni,d_, and *ω*_ni,m_ were determined to be 0.3793, 0.0042 and 0.4470, respectively. The fact that *ω*_i,d_ ≫ *ω*_ni,d_ indicates that the dimer solution is comprised mostly of dimers. Although the results suggest that the technique is able to provide a quantitative estimate of the relative concentration of probe and dimer (as our interacting model) populations, we have found it difficult to ascertain a suitable technique for accurate comparison. DLS was not capable of providing robust estimates and appeared to provide a monomer peak of reduced size (by 4 nm) for the dimer sample. Aggregates (>100 nm) were detected when plotting the size distribution by intensity (as opposed to number %), not visible under SAXS. The size of the aggregates were beyond the measurable particle dimension of the SAXS configuration used.

**Figure 6:**
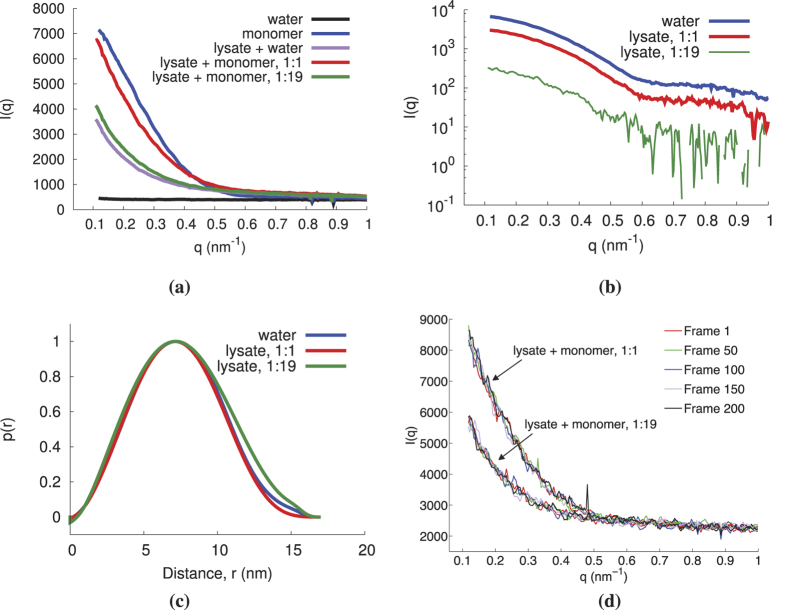
The scattering signal of monomers in cell lysate and water backgrounds were compared, where the monomer in lysate was tested for two dilutions, 1:1 and 1:19. (**a**) The dark-subtracted scattering intensity of monomers and their respective background. (**b**) The subtracted scatter curves of the monomers in water and lysate. (**c**) The *p*(*r*) of monomers in water and lysate. The monomers in lysate and water show good agreement. Only slight deviations in *p*(*r*) are seen for the low concentration monomer (1:19) in lysate. (**d**) Scatter curves (not background subtracted) for different frames (frames 1, 50, 100, 150, and 200) throughout the exposure time. No trend is observed with increasing frame, suggesting no significant damages have occured to the sample.

We then tested whether or not spurious background scattering from other molecules typical of a cellular environment would significantly hinder the method’s performance by measuring the model system in the presence of a cell lysate. The scattering signal from probes in lysate and in water was measured to confirm that the probes could be identified (Fig. 6). Because DTT, the reducing agent present in the lysis buffer, contains a thiol moiety and is present in high relative concentrations, it readily competes with the PEG coating, causing the breaking of the dimer linker^51^ and the inability to observe dimers when mixed with the lysis buffer. In order to determine if the effects observed in the presence of DTT are due to the thiol moiety or its reducing capacity, as well as to confirm that scattering signatures could be observed when the GNPs are in the lysate solution, similar experiments were conducted using TCEP (tris(2-carboxyethyl)phosphine). The reducing agent TCEP contains a phosphine moiety which does not compete with the PEG linker-thiol allowing for the dimer to remain intact in the lysate suspension (data not shown).

**Figure 7:**
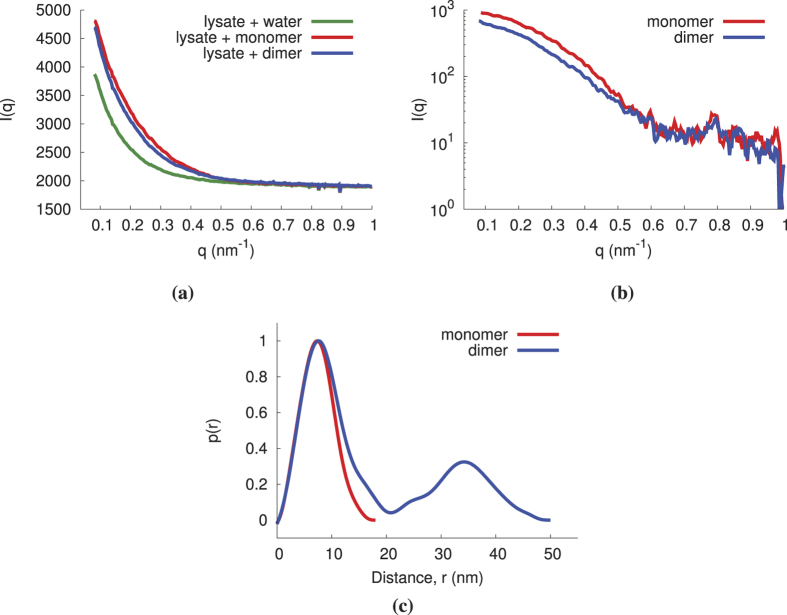
The scattering signal of monomers and dimers with a cell lysate background (separate capillary). (**a**) The dark-subtracted scattering intensity of a monomer and dimer solution and their background signal. (**b**) The subtracted scatter curves of the monomer and dimer solutions. (**c**) The *p*(*r*) of the monomer and dimer solutions.

In light of this, we placed a separate adjacent capillary holding the lysate in the primary X-ray beam path in order to simulate lysate noise without compromising the integrity of the probes. The results confirm that the technique is capable of detecting the presence of the interacting species model even in the presence of cell lysate scatter noise (Fig. 7).

## Discussion

In this work, we demonstrate that detection and characterization of interacting species labelled with GNPs are feasible with our SAXS-based technique. Although the model system was measured as a stationary solution inside a quartz microcapillary, the method has potential for three dimensional imaging and dynamic studies. For example, the use of a scanning beam and/or rotating mechanism would allow for 2D and 3D imaging^52^. In this sense, the described method will have applications in medicine, potentially allowing 3D imaging of biological process associated with disease at the cellular level, in small animals, and in human subjects. Dynamic (i.e., time-resolved) studies are also possible with X-ray sources that provide sufficient brilliance and, if necessary, flow samples^53^. To reduce detector noise an energy-discriminating or photon-counting detector could be used as opposed to an energy integrating detector as used in this study. An accelarator source could also be used to improve data quality.

Using SAXS to study biomolecular interactions enables high spatial resolution characterization of the interaction itself (through *p*(*r*), *ω*_i_(*s*), and the spatial model). Our method differs from BRET and FRET in that there is superior control over the probe size and interactions for systems larger than 10 nm. The spatial resolution in terms of localization is limited by the beam size, which is on the order of 300 *μ*m (pinhole diameter) for laboratory systems and 25 *μ*m for accelerator instrumentation^52^.

The addition of SAXS to the toolbox of methods used to complete PPI databases can significantly expand existing knowledge of PPIs while improving the quality of known PPI data by means of validation of previously contrived data. Ongoing work aims at adding SAXS data to the global repository of PPIs in a new database tool for the creation of a protein network interface that would allow users to create PPI hypotheses based on shared participation of proteins in similar biological networks, while supplementing empirically and experimentally derived protein interaction data from existing databases^54^.

In summary, we report a novel method for characterizing interactions among labelled species from the scattering signature of highly scattering molecular probes. From the scattering signature, information on the interaction fraction and high resolution spatial information (i.e., interaction distance) can be obtained. The method can be realized with a bench-top SAXS system, as demonstrated herein, or with an accelerator, as long as low *q* values corresponding to biomolecular interaction distances are resolvable by the instrumentation (lower resolvable *q* values correspond to larger resolvable distances). Although challenges remain in binding probes to targeted molecules, with sufficiently high scattering signals from optimally engineered molecular probes, the method has potential for applications *in vitro*, *in cellulo*, and *in vivo*.

## Additional Information

**How to cite this article**: Allec, N. *et al.* Small-angle X-ray scattering method to characterize molecular interactions: Proof of concept. *Sci. Rep.* **5**, 12085; doi: 10.1038/srep12085 (2015).
